# Association between body mass index and posterior spine fusion among patients with adolescent idiopathic scoliosis

**DOI:** 10.1371/journal.pone.0286001

**Published:** 2023-05-18

**Authors:** Carrie T. Chan

**Affiliations:** 1 Stanford Medicine Children’s Health, Palo Alto, California, United States of America; 2 Department of Epidemiology and Biostatistics, University of California, San Francisco, San Francisco, California, United States of America; 3 Department of Family Health Care Nursing, University of California, San Francisco, San Francisco, California, United States of America; Emory University School of Medicine, UNITED STATES

## Abstract

**Introduction:**

Previous studies have found mixed associations between body mass index (BMI) and adolescent idiopathic scoliosis (AIS) incidence and progression. The aim of this study was to examine the association between BMI and the incidence of posterior spine fusion (PSF) among pediatric patients with AIS.

**Methods:**

This was a retrospective cohort study of patients diagnosed with AIS at a single large tertiary care center between January 1, 2014 and December 31, 2020. BMI-for-age percentiles were used to categorize BMI into four categories: underweight (<5th percentile), healthy weight (≥5th to <85th percentile), overweight (≥85th to <95th percentile), and obese (≥95th percentile). Chi-square and t-tests were used to compare distributions of baseline characteristics by incident PSF outcome status. Multivariable logistic regression assessed the association between BMI category at baseline and incident PSF adjusting for sex, age at diagnosis, race/ethnicity, health insurance type, vitamin D supplementation, and low vitamin D levels.

**Results:**

A total of 2,258 patients met the inclusion criteria with 2,113 patients (93.6%) who did not undergo PSF during the study period and 145 patients (6.4%) who did undergo PSF. At baseline, 7.3% of patients were categorized as underweight, 73.2% were healthy weight, 10.2% were overweight, and 9.3% were obese. Compared to those in the healthy weight group, there was no significant association between PSF and being underweight (adjusted odds ratio [AOR] 1.64, 95% CI 0.90–2.99, p = 0.107), being overweight (AOR 1.25, 95% CI 0.71–2.20, p = 0.436), or being obese (AOR 1.19, 95% CI 0.63–2.27, p = 0.594).

**Conclusions:**

This study did not find a statistically significant association between underweight, overweight, or obese BMI category and incident PSF among patients with AIS. These findings add to the current mixed evidence on the relationship between BMI and surgical risk and may support the recommendation of conservative treatment to patients regardless of BMI.

## Introduction

Adolescent idiopathic scoliosis (AIS) is the most common type of pediatric spine deformity with a prevalence of up to 5% in adolescents 18 years and younger [1]. Among adolescents with AIS, approximately 10% have progressive AIS that worsen with skeletal growth, requiring treatment with bracing and sometimes invasive spine fusion surgery to prevent continued progression and disability in adulthood [2]. In the United States, approximately 38,000 pediatric patients undergo spine fusion each year for progressive scoliosis [3].

There is little known about the causes and predictors of progressive AIS. There have been few identified modifiable risk factors that have been found to be clinically relevant to prevent progression [4, 5]. Factors such as BMI, vitamin D levels and supplementation, high-risk sports participation, and posture disorders have previously been studied with no clear, strongly established relationships [6–15].

Mixed associations have been found between body mass index (BMI) and AIS in pediatric patients. Some studies have found that lower BMI is associated with scoliosis diagnosis and increased curve severity whereas others have found that overweight patients present with larger scoliosis curves and that higher adiposity is significantly related to more severe scoliosis [6–8]. Studies looking at conservative treatment outcomes also found inconsistent results; one found that overweight patients have greater curve progression despite treatment [9], one found that underweight patients had the highest risk of progressing to severe curves [10], one found that both high and low BMI were more likely to fail brace treatment than mid-BMI patients [11], and still another found no difference between overweight and normal weight patients [12]. Additionally, the biological mechanisms behind the relationship between BMI and AIS are largely unknown [16]. Previous literature includes hypothesized multifactorial mechanisms such as genetic, biomechanical, and environmental factors as well as hormonal and metabolic dysfunctions [16]. However, there remains considerable variability in the literature, and etiopathogenesis of AIS and the relationship and mechanism between BMI and progressive AIS that requires surgical intervention remain largely unknown [16].

The aim of this study was to examine the association between BMI and the incidence of posterior spine fusion among pediatric patients with AIS using a large electronic health record (EHR) dataset.

## Materials and methods

### Study design and patient selection

This was a retrospective cohort study of patients at a single large tertiary care center in the United States with the largest network of pediatric providers in the geographic region and that is one of only two centers in that region with specialized, dedicated pediatric orthopedic care. Patients diagnosed with AIS between January 1, 2014 and December 31, 2020 were included. Follow-up patient clinical data through the age of 25 or April 30, 2022 (whichever was earlier) was utilized for this study from the center’s de-identified EHR clinical data warehouse. Patients eligible for inclusion were pediatric patients with a diagnosis consistent with AIS (ICD-9 737.30, ICD-10 group M41.1, ICD-10 group M41.2), initial diagnosis age between 10 and 18 years inclusive, and initial diagnosis date between January 1, 2014 and December 31, 2020. Patients who did not meet the inclusion criteria listed above, who did not have at least one encounter in the EHR clinical data warehouse during the study period, or who did not have available BMI measurements were excluded.

### Explanatory and independent variables

The primary predictor in this study was baseline BMI, which was available as a continuous variable in the clinical data warehouse. BMI was converted to BMI-for-age percentiles according to Centers for Disease Control and Prevention clinical growth charts [17]. BMI-for-age percentiles were then used to categorize BMI into four weight status categories: underweight (<5th percentile), healthy weight (≥5th to <85th percentile), overweight (≥85th to <95th percentile), and obese (≥95th percentile). Baseline BMI was defined as earliest recorded BMI at or after first recorded diagnosis of scoliosis. If BMI on the day of first recorded scoliosis diagnosis was not available, the next available BMI measurement was used.

Baseline patient characteristics considered as confounding variables that have a known influence on both patient baseline BMI and incident posterior spine fusion included biological sex [18], age at first recorded diagnosis of scoliosis [18], race/ethnicity [19], and health insurance status [20, 21]. Vitamin D supplementation and vitamin D levels, which are hypothesized predictors of the outcome of incident spine fusion [14, 15], were also included to potentially improve statistical precision. Biological sex was coded as a dichotomous variable limited to available EHR response options of male and female. Age at first recorded scoliosis diagnosis was a continuous variable in years. Race/ethnicity as identified in the patient medical record was coded categorically limited to White, Latinx, Asian, Black/African American, Native Hawaiian/Pacific Islander, American Indian/Alaska Native, other, and unknown. Health insurance was coded dichotomously as private insurance or government insurance/unknown. Vitamin D supplementation was coded as a dichotomous variable indicating if a patient was on vitamin D supplementation at any point during the study period. Low vitamin D was coded as a dichotomous variable indicating if a patient had a recorded low vitamin D level during the study period.

### Outcomes

The outcome of interest in this study was incident posterior spine fusion with instrumentation at or before the age of 25 as treatment for progressive, severe AIS. This was defined as a dichotomous outcome based on patient encounters with CPT codes 22802 or 22804 during the study period.

### Statistical methods

Frequency analyses were conducted to describe baseline participant characteristics. Chi-square and t-tests were used to compare distributions of baseline characteristics by incident posterior spine fusion outcome status. Multivariable logistic regression assessed the association between BMI category at baseline and incident posterior spine fusion adjusting for sex, age at diagnosis, race/ethnicity, health insurance type, vitamin D supplementation, and low vitamin D levels.

A sensitivity analysis was done by including variables accounting for the amount of time between scoliosis diagnosis and first BMI measurement as well as total amount of follow-up time (from scoliosis diagnosis to either surgery for those who underwent spine fusion or last documented encounter for those who did not). This was done to evaluate for any change in results due to potential delay between first diagnosis and BMI measurement and variable follow-up time among patients.

An additional sensitivity analysis was done limiting the analysis to only patients with BMI measurements available at the time of first documented scoliosis diagnosis. This was done to check for any change in results due to using the next available BMI measurement as a proxy for baseline BMI in patients without available BMI data at time of documented scoliosis diagnosis.

Statistical analyses were done using Stata 15.1 [College Station, TX: StataCorp LLC].

## Results

A total of 3,739,302 patients were assessed for inclusion. After excluding patients who did not meet the inclusion criteria or who did not have BMI data available, 2,258 patients were included (Table 1) with 2,113 patients (93.6%) who did not undergo spine fusion during the study period and 145 patients (6.4%) who did undergo spine fusion. Most patients in the total sample were female (65.7%), and an even larger proportion of patients who underwent spine fusion were female (82.1%). The mean diagnosis age among the total population was 14 years (standard deviation 2.0 years). Patients most commonly identified as either White (39.6%) or Latinx (25.9%). A majority of patients were covered by private insurance (56.6%). A small proportion of total patients had documented vitamin D supplementation (8.46%) or low vitamin D levels (9.92%).

**Table 1:** Descriptive statistics and bivariate analyses of study population baseline characteristics by spine fusion outcome.

Participant Characteristics		Total (N = 2,258)		No spine fusion (N = 2,113)		Spine fusion (N = 145)		p-value
Sex, N(%)								
	Female	1,484	(65.72)	1,365	(64.60)	119	(82.07)	<0.001
	Male	774	(34.28)	748	(35.40)	26	(17.93)	
Age at diagnosis, years (SD)		13.89	(2.03)	13.89	(2.05)	13.83	(1.84)	0.738
Race/ethnicity, N(%)								
	White	893	(39.55)	830	(39.28)	63	(43.45)	0.001
	Latinx	585	(25.91)	562	(26.60)	23	(15.86)	
	Asian	247	(10.94)	223	(10.55)	24	(16.55)	
	Other	198	(8.77)	182	(8.61)	16	(11.03)	
	Black/African American	150	(6.64)	145	(6.86)	5	(3.45)	
	Multi-race/ethnicity	50	(2.21)	42	(1.99)	8	(5.52)	
	Native Hawaiian/Pacific Islander	19	(0.84)	18	(0.85)	1	(0.69)	
	American Indian/Alaska Native	11	(0.49)	9	(0.43)	2	(1.38)	
	Unknown	105	(4.65)	102	(4.83)	3	(2.07)	
Health insurance type, N(%)								
	Private	1,279	(56.64)	1,184	(56.03)	95	(65.52)	0.026
	Government/unknown	979	(43.36)	929	(43.97)	50	(34.48)	
BMI baseline, N(%)								
	Underweight	165	(7.31)	151	(7.15)	14	(9.66)	0.676
	Normal	1,653	(73.21)	1,550	(73.36)	103	(71.03)	
	Overweight	231	(10.23)	215	(10.18)	16	(11.03)	
	Obese	209	(9.26)	197	(9.32)	12	(8.28)	
Vitamin D supplementation, N(%)								
	Yes	191	(8.46)	176	(8.33)	15	(10.34)	0.399
	No	2,067	(91.54)	1,937	(91.67)	130	(89.66)	
Low vitamin D, N(%)								
	Yes	224	(9.92)	206	(9.75)	18	(12.41)	0.299
	No	2,034	(90.08)	1,907	(90.25)	127	(87.59)	
Provider specialty, N(%)								
	Orthopedic surgery	1,413	(62.58)	128	(88.28)	1,285	(60.81)	<0.001
	Primary care	259	(11.47)	2	(1.38)	257	(12.16)	
	Other specialty	586	(25.95)	15	(10.34)	571	(27.02)	
Time between diagnosis and BMI measurement, months (SD)		9.13	(16.43)	9.51	(16.77)	3.60	(8.35)	<0.001
Follow-up time^a^, months (SD)		21.60	(22.52)	22.18	(22.87)	13.17	(14.13)	<0.001

Note: SD = standard deviation; BMI = body mass index

^a^ Time between first diagnosis and last available BMI measurement among those without surgery or surgical date among those with surgery

At baseline, 7.3% of patients were categorized as underweight, 73.2% were healthy weight, 10.2% were overweight, and 9.3% were obese. This BMI distribution seen in the total population did not differ significantly from the proportion of patients in each BMI category in the outcome groups of no spine fusion and spine fusion.

### Univariable analysis

Table 2 shows that the crude logistic regression model (Model 1) demonstrated no significant overall association between any BMI category and incident posterior spine fusion (p = 0.679). Specifically, compared to those in the healthy weight group, there was no significant association between surgical intervention and being underweight (odds ratio [OR] 1.40, 95% confidence interval [CI] 0.78–2.50, p = 0.263), being overweight (OR 1.12, 95% CI 0.65–1.93, p = 0.684), or being obese (OR 0.92, 95% CI 0.50–1.70, p = 0.782).

**Table 2:** Associations between body mass index and posterior spine fusion with crude and adjusted logistic regression models.

	Model 1^a^			Model 2^b^		
	OR	95% CI	p-value	AOR	95% CI	p-value
Underweight	1.40	(0.78, 2.50)	0.263	1.64	(0.90, 2.99)	0.107
Healthy weight	Ref	—	—	Ref	—	—
Overweight	1.12	(0.65, 1.93)	0.684	1.25	(0.71, 2.20)	0.436
Obese	0.92	(0.50, 1.70)	0.782	1.19	(0.63, 2.27)	0.594
Total^c^			0.679			0.391

Note: OR = odds ratio; AOR = adjusted odds ratio; CI = confidence interval

^a^ Bivariate logistic regression model

^b^ Multivariable logistic regression model adjusting for sex, age at diagnosis, race/ethnicity, health insurance type, vitamin D supplementation, low vitamin D

^c^ Wald chi-square test

### Multivariable analysis

The multivariable regression model (Table 2, Model 2) adjusting for biological sex, age at diagnosis, race/ethnicity, health insurance type, vitamin D supplementation, and low vitamin D levels also demonstrated no significant overall association between BMI and incident spine fusion (p = 0.391). Specifically, compared to those in the healthy weight group, there was no significant association between surgical intervention and being underweight adjusting for sex, age at diagnosis, race/ethnicity, health insurance type, vitamin D supplementation, and low vitamin D levels (adjusted odds ratio [AOR] 1.64, 95% CI 0.90–2.99, p = 0.107), being overweight (AOR 1.25, 95% CI 0.71–2.20, p = 0.436), or being obese (AOR 1.19, 95% CI 0.63–2.27, p = 0.594).

### Sensitivity analyses

The model including variables for the amount of time between diagnosis and BMI measurement as well as total follow-up time per patient demonstrated no notable difference in results compared to the primary adjusted analysis (Table 3, Model 3).

**Table 3:** Sensitivity analyses adjusting for time between scoliosis diagnosis and first available body mass index measurement as well as follow-up time per patient.

	Model 3^a^			Model 4^b^			Model 5^c^		
	AOR	95% CI	p-value	OR	95% CI	p-value	AOR	95% CI	p-value
Underweight	1.75	(0.95–3.23)	0.072	1.42	(0.65, 3.07)	0.377	2.03	(0.91, 4.53)	0.085
Healthy weight	Ref	—	—	Ref	—	—	Ref	—	—
Overweight	1.29	(0.73–2.28)	0.384	1.28	(0.62, 2.67)	0.505	1.32	(0.62, 2.81)	0.470
Obese	1.12	(0.58–2.16)	0.729	1.34	(0.62, 2.90)	0.457	1.77	(0.77, 4.07)	0.182
Total^d^			0.302			0.691			0.220

Note: AOR = adjusted odds ratio; OR = odds ratio; CI = confidence interval

^a^ Multivariable logistic regression model adjusting for sex, age at diagnosis, race/ethnicity, health insurance type, vitamin D supplementation, low vitamin D, time between diagnosis and BMI measurement, follow-up time

^b^ Bivariate logistic regression model limited to patients with BMI measurement on same date as first documented scoliosis diagnosis

^c^ Multivariable logistic regression model adjusting for sex, age at diagnosis, race/ethnicity, health insurance type, vitamin D supplementation, low vitamin D limited to patients with BMI measurement on same date as first documented scoliosis diagnosis

^d^ Wald chi-square test

Limiting the crude (Table 3, Model 4) and multivariable logistic regression analyses (Table 3, Model 5) only to patients with available BMI measurements at time of first recorded scoliosis diagnosis, the total sample size was 1,127 patients with 1,037 (92.0%) who did not undergo spine fusion and 90 patients (8.0%) who did receive surgical intervention (Table 3). This demonstrated the same results as the primary analysis for both crude and adjusted logistic regression models.

## Discussion

This study found that having a baseline BMI consistent with being underweight, overweight, or obese was not associated with increased odds of undergoing posterior spine fusion compared to being categorized as healthy weight among patients with AIS. This was consistent in both crude and adjusted analyses.

These findings contribute to the current mixed evidence on this topic. The results from this study are in contrast with previous studies that found that lower BMI [6], higher BMI [8, 9], or both [10, 11] were associated with scoliosis curve severity, failure of conservative brace treatment, and risk for surgery. This study’s findings are consistent with prior literature that found that participants who were overweight were not more likely to fail conservative treatment and experience curve progression [12]. This is important to consider, as previous studies have suggested that patient BMI should be taken into account when making treatment decisions [9]. Our findings instead suggest that having a BMI categorized as underweight, overweight, or obese does not have a statistically significant association with undergoing spinal fusion; therefore, BMI perhaps should not be a consideration when deciding whether or not to initiate conservative treatment for AIS. Instead, conservative treatment recommendations should be offered and supported consistent with current bracing evidence for curve magnitude and stage of skeletal maturity regardless of patient BMI at time of scoliosis diagnosis.

In fact, it may be even more important to support patients with BMI ≥ 85th percentile to be adherent to evidence-based bracing and conservative treatment given that previous studies have been consistent in their findings that overweight and obese patients have higher rates of surgical complications [22–25]. This includes intraoperative complications such as increased surgical times and blood loss and postoperative complications including longer hospital stays, surgical site infections, would complications, readmissions, and reoperations [22–25].

Strengths of this study include the large sample size of patients with AIS and the inclusion of multiple statistical models and sensitivity analyses that were consistent in their findings. There are also several limitations to this study that warrant consideration. Because EHR data was used for this study, data quality is limited to the accuracy of clinical data entry in the EHR. For example, date of scoliosis diagnosis for this study was the first date that an ICD-9 or ICD-10 code consistent with AIS was entered into this institution’s EHR; this may not be consistent with the actual first date of diagnosis if scoliosis was initially diagnosed at an outside institution or a different code was initially entered. Information on family history of scoliosis, which is considered a confounder, was not available in the EHR, so it remains possible that our associations were affected by this and other unmeasured variables. Similarly, information regarding baseline scoliosis curve magnitude and conservative treatment with bracing, which are considered mediators, were not available in the deidentified EHR data; omitting these variables resulted in estimations of total effect including any indirect effects from these variables. Future directions to follow up on this study include building models that can further study the direct effect of BMI on need for spine fusion by including important mediators such as curve magnitude at diagnosis and conservative treatment using unstructured clinic and radiology note data. Additionally, approximately 50% of the study sample did not have a BMI measurement available on the same date as the first recorded scoliosis diagnosis. However, sensitivity analyses demonstrated no change in results when adjusting for this delay or limiting the analysis to only patients with available BMI measurement on the same date as first recorded scoliosis diagnosis. This study also assumes that among the study population of patients diagnosed with AIS, if a patient required surgery, they received their fusion internally within the institution. While undergoing spine fusion is an indicator of severe scoliosis, there is oftentimes a delay between surgery indication and surgery itself. This was a single-institution study, so generalizability may be limited.

This study did not find a statistically significant association between underweight, overweight, or obese BMI category and incident posterior spine fusion among patients with AIS. These findings add to the current mixed evidence on the relationship between BMI and surgical risk and may support the recommendation of conservative treatment to patients regardless of BMI. However, given the mixed evidence on the association between BMI and progressive scoliosis, larger studies or meta-analyses are needed to better understand the relationship between BMI and potential need for spine fusion. Further research should be done to determine any potential causal effect and also mechanism of the relationship between BMI and progressive scoliosis.
